# Slow-Speed Resistance Training Increases Skeletal Muscle Contractile Properties and Power Production Capacity in Elite Futsal Players

**DOI:** 10.3389/fspor.2020.00008

**Published:** 2020-02-07

**Authors:** Pierpaolo Iodice, Athos Trecroci, Dario Dian, Giorgia Proietti, Giampietro Alberti, Damiano Formenti

**Affiliations:** ^1^CETAPS—EA 3832, University of Rouen Normandy, Mont-Saint-Aignan, France; ^2^Institute of Cognitive Sciences and Technologies—Consiglio Nazionale delle Ricerche, Rome, Italy; ^3^Department of Biomedical Sciences for Health, Università degli Studi di Milano, Milan, Italy; ^4^Faculty of Sport Sciences, University of Chieti, Chieti, Italy; ^5^Department of Biotechnology and Life Sciences (DBSV), University of Insubria, Varese, Italy

**Keywords:** team-sport, elite athletes, strength training, sport, isokinetic, force

## Abstract

The purpose of this study was to explore the effects of slow-speed resistance training with low intensity (SRT) on muscle power output in elite futsal players with respect to traditional resistance training. The authors hypothesized that the muscle deoxygenation during SRT causes early recruitment of fast twitch fibers that would positively affect strength and power performance. Thirty male elite futsal players were recruited and randomly assigned either to SRT group or to traditional resistance training (TRT) group. All players underwent an 8-weeks experimental protocol consisting of 2 training sessions per week at both leg curl and leg extension machines. In the SRT, players lifted 50% of one maximum repetition (1RM) involving 3 s for eccentric and concentric actions. In the TRT, players lifted 80% of 1RM involving 1 s for eccentric and concentric actions. All players were tested twice (pre and post) for sprint and jump performances, maximal isometric voluntary contraction (MVC) and maximal isokinetic peak torque (Peak TQ) and total work (TW) at 60 and 120°/s (on knee extensors and flexors). The two groups presented remarkable differences in the within-group changes for all the variables. SRT exhibited greater improvements in both Peak TQ and TW for knee extensors and flexors at 120°/s. Conversely, TRT showed greater improvements in MVC, and in both Peak TQ and TW for knee extensors and flexors at 60°/s, except for Peak TQ of the knee extensors, where no significant difference was found between TRT and SRT. Countermovement jump showed a decrease in eccentric time and an increase in concentric force in SRT group. SRT and TRT resulted effective to enhance the strength performance indices during the 8-weeks experimental protocol. Peak torque at 120°/s explained more of the contractile characteristic effects of SRT training than MVC, suggesting that slow-speed training can cause fast twitch fibers hypertrophy in elite athletes. Since slow-speed training is supposed to produce a decreased exercise-induced muscle damage, SRT method is a suitable option in strength training for futsal and team sports.

## Introduction

In team sports, training methods are usually based on the specificity principle, which aims to recreate many factors of a sport performance, such as: principal contraction type, distance covered, characteristic movements, and main energy consumption sources (Bangsbo et al., 2006). Nevertheless, there is unanimous consensus that strength and power capacity are critical physical determinants for success in team sports. Therefore, resistance sessions are an essential part of athletes training (Hoff and Helgerud, 2004). The more commonly used resistance training method is based on high-intensity (~80% of 1 maximal repetition, 1RM). This methodology was found to be effective for increasing strength-related variables (i.e., power) in soccer players (Hoff and Helgerud, 2004). It has been shown, however, that resistance exercise using high-intensity or loads causes delayed strength recovery due to muscle damage (Ahtiainen et al., 2003). Moreover, studies demonstrated that faster speed of movement induced greater delayed onset muscle soreness (DOMS) (Kulig et al., 2001) as well as Z-band disruption (Shepstone et al., 2005). These factors lead to long-lasting impairments in motor performance, thereby restricting the use of high-intensity resistance training within the weekly routine of team sports athletes.

For this reason, several studies within the field of sports sciences have been conducted to identify a training method able to increase muscle strength while reducing the damage related to strength training. Among others, studies have investigated the effects of slow-speed movement and low-intensity resistance training (SRT) (Tanimoto and Ishii, 2006; Tanimoto et al., 2008, 2009). It was found that muscle size and strength improved similarly in both SRT and high intensity resistance training (Tanimoto and Ishii, 2006; Tanimoto et al., 2008, 2009). The authors suggested that SRT, by prolonged muscle contraction time, induced muscle deoxygenation (Tanimoto and Ishii, 2006), as obtained by blood flow restriction training, without the application of a cuff device (Ganesan et al., 2015). In support of this notion, a previous study showed similar muscle deoxygenation during blood flow restriction resistance training and slow movement low intensity resistance training (Tanimoto et al., 2005).

To the best of our knowledge, the effect of SRT has not been investigated in elite team athletes, but almost exclusively on recreational (Tanimoto and Ishii, 2006) or elderly subjects (Watanabe et al., 2013, 2015). The main reason is related to the specificity of the muscle contraction velocity, which falls outside of field-based team sports demands (high stretch-shortening contraction) (Trecroci et al., 2016). In SRT, the speed of movement is intentionally kept slow, thus suppressing inertia and inducing a continuous muscular tension throughout the exercise (Tanimoto et al., 2008). These have always been considered unfavorable effects on sports-related movement tasks, and consequently, on physical performance. In fact, in accordance with the Henneman's principle (Henneman et al., 1965), only high-intensity resistance training can cause sustained activation of fast-twitch fibers, whereas slow-twitch fibers are the most recruited during low-intensity resistance training.

Recently, Husmann et al. (2017) have shown that a reduced muscular oxygenation, induced by a restriction of muscular blood flow, evokes early recruitment of fast twitch fibers (Husmann et al., 2017). These findings, and the evidence that SRT induces muscular deoxygenation (Tanimoto and Ishii, 2006), lead us to hypothesize that slow training can stimulate the fast twitch fibers even through quite low-intensity. The slow training method (i.e., 3/0/3/0) employed in these studies reduces peripheral muscular oxygenation during exercise (Formenti et al., 2018, 2019). Moreover, a previous study reported that slow-speed resistance exercise induced low muscle damage compared with faster speed (Paddon-Jones et al., 2005; Barroso et al., 2010; Chapman et al., 2011).

On the other hand, given the lower impact on muscle damage and the likely effect on the recruitment of fast twitch fibers, SRT can be considered a suitable method not only for the elderly (Watanabe et al., 2013), but also for team athletes (Alberti et al., 2013). This notion is particularly important for training periodization of team sports, because of the reduced recovery time available between a match and the subsequent training session (Bangsbo et al., 2006).

Therefore, the aim of the present study was to investigate the effects of SRT on strength-related and neuromuscular performance with respect to traditional resistance training in elite futsal players. We hypothesized that low-intensity resistance training (~50% of 1RM) with reduced muscular oxygenation, caused by slow-speed movement, would affect positively muscle power, jump and sprint performance.

## Materials and Methods

### Participants

Thirty male elite futsal players (age, 24 ± 1.5 year; height, 1.73 ± 0.11 month; body mass, 67.1 ± 9.2 kg; body mass index, 20.26 ± 3.11 kg m^−2^; maximal oxygen uptake, 57.4 ± 6.5 ml kg^−1^ min^−1^) voluntarily participated in this study.

This study is part of a project designed to investigate the dynamics between fatigue and cognitive skills. The project was approved by the Ethics Committee of Institute of cognitive sciences and technologies, CNR, of Rome, Italy (N° 0003871–24/12/2015). The study was performed in accordance with the ethical standards of the Declaration of Helsinki. The participants received the explanations of the procedures, as well as the risks and discomforts involved in the study and signed the written consent form. The participants were asked to refrain from any resistance exercise during the whole experimental period. Moreover, they were asked to abstain from drinking caffeinated beverages for 48 h prior to testing.

### Experimental Design

**Figure 1:**
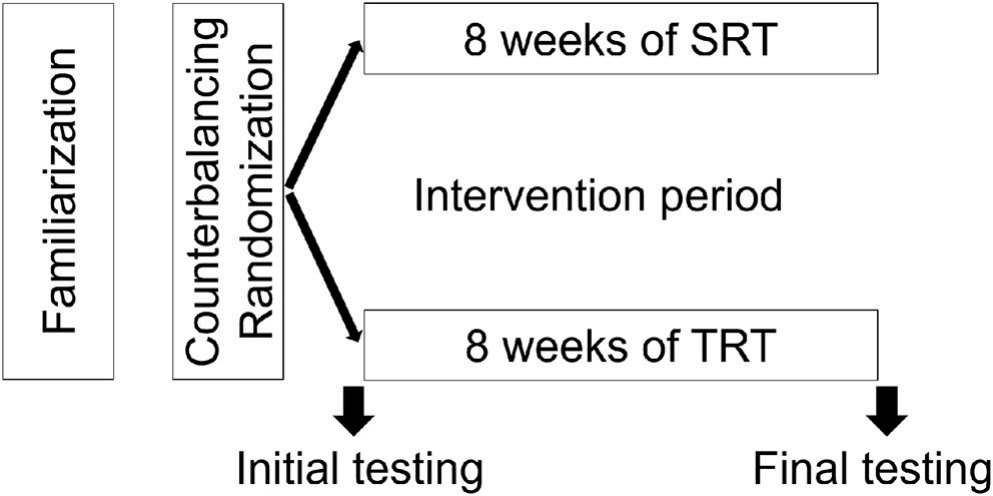
Scheme of the experimental design. The two testing sessions (initial and final) included the assessment of 30 m sprint, squat jump, countermovement jump, maximal isometric voluntary contraction of knee extensors, and maximal isokinetic peak torque and total work of knee extensors and flexors. SRT, Slow speed resistance training; TRT, Traditional resistance training.

The study was conducted during the competitive period with a weekly training routine based on 5 days of training sessions and one match play. Participants were counterbalanced on pre-test maximal voluntary contraction values and randomly assigned to slow resistance training (SRT, *n* = 15) and traditional resistance training (TRT, *n* = 15) groups. The training intervention consisted of 8 weeks with two sessions per week (see Figure 1 for experimental design flowchart). Each subject underwent two testing sessions before and after training intervention. All testing sessions were administered in the late morning to mitigate possible effects related to circadian rhythm variation. On the first testing day, after anthropometric measurements recording, participants performed the sprint tests over 30 and 60 m and countermovement jump test (CMJ). Moreover, participants performed the 1RM test at the leg extension and leg curl machines using the protocol described by Maud and Foster (2006). On the second testing day (after 96 h) the participants performed the maximum voluntary contraction (MVC) and knee extensors concentric isokinetic test for both knee flexors and extensors at two angular velocities (i.e., 60 and 120°/s). Prior to each testing session, the participants warmed up for 10 min on a cycle ergometer with an intensity of 50 W with a constant pedaling rate (i.e., ~90 rpm).

All tests were executed randomly with 10 min of recovery in between (Di Pancrazio et al., 2013).

### Training Protocol

The participants in each training group performed bilateral leg extension and leg curl exercises on the corresponding isotonic machines (Panatta srl, Apiro, Italy) in seated position. During the experimental exercises, the range of joint motion was controlled and kept from 0 to 90° (0° at full extension). The participants of SRT performed three sets of low intensity exercise (50% of 1RM); with slow movement (3/0/3/0, 3-s eccentric phase, no break in the transition phase, a 3-s concentric phase and no rest before the next repetition); interspersed by 60 s of rest for both exercises (i.e., leg extension and leg curl). The participants were required to exercise until exhaustion for each set. During the exercise, a certified operator controlled the movement tempo (cadence) recording all sessions through a camera (Hatfield et al., 2006; Wilk et al., 2018). Time under tension (TUT) for the whole training was calculated by longomatch software analysis (https://longomatch.com) at the end of each session. TUT was monitored session by session throughout the 8-weeks training program. With the aim of having the same TUT for both groups, the TRT training protocol was designed to correspond approximately to the TUT recorded during SRT for each training session.

During the 8-weeks TRT protocol, participants initially performed 6 sets of 8 high intensity (80% of 1RM) repetitions with normal speed of movement (1/0/1/0) interspersed by 60 s of rest for both exercises. After 5 min of rest, the athletes continued their training until they reached the same volume and intensity of TUT carried out by the STR group; bearing in mind that 1 s under tension at 50% 1RM equals to 0.62 s under tension at 80% 1RM. For example: a set of 10 repetitions at 50% 1RM performed with the slow training method (e.g., TUT50 = 60 s) was considered equivalent to three sets of six repetitions at 80% 1RM (e.g., TUT80 = 36 s then TUT50 = 58 s). The TRT protocol was adjusted by the corresponding TUT of SRT to standardize volume and intensity ratio session by session. A warm up consisting of 5 min of low intensity (based on running drills and dynamic stretching) was performed at the beginning of each training session.

### Maximum Voluntary Contraction (MVC)

Isometric strength developed by knee extensor muscles was measured during maximal voluntary contractions using a leg extension machine (Panatta Sport; Apiro MC, Italy) equipped with a load cell (rating from 10 N to 5 kN, Globus^®^, Codogné, Italy). Participants were seated with the trunk-thigh and the knee joint angles at 90°. Each subject was tested on MVC three times. The participants were instructed to perform isometric contractions lasted for 5 s separated by 1 min of rest. The highest value of torque (i.e., peak torque) was taken as the MVC (Saggini et al., 2009).

### Isokinetic Assessment

Knee flexors and extensors muscle torque was determined on the dominant limb at 60°/s and 120°/s between 90° and 20° (0° = full knee extension) on an isokinetic dynamometer (Cybex International Inc., Medway, MA) with participants in seated position. The distal pad of the dynamometer arm was placed in the proximity of the malleoli. The axis of rotation of the dynamometer was adjusted so that the device was aligned with the joint margin of the knee. Fifteen warm-up trials and preconditioning of the testing device were performed prior to data sampling at 45% of subjective maximal effort. Participants performed three repetitions during which the highest peak torque (Peak TQ) (N·m) and the total work (TW) (J) produced by flexors and extensors muscle (Iodice et al., 2015) were considered for the subsequent analysis.

### Jump Tests

All participants performed three maximal bouts of countermovement jumps (CMJ) on a force platform (Globus^®^, Codogné, Italy) with their hands on the hips. A 2-min rest period was given between jump tests. The knee angular displacement (i.e., at 90°) was standardized by an electronic goniometer. The jump height (cm) was determined as the center of mass displacement calculated from force-time characteristics and body mass (Iodice et al., 2011).

The independent variables for CMJ were extracted from the force-time curve. The eccentric rate of force development (ECC-RFD) was based on the average slope of the eccentric loading portion of the force/time curve; it begins when force exceeds body weight and ends when velocity comes to zero. This variable has been normalized by body mass (R-ECC-RFD) to study the independent mass effect. TIME is the total time of the CMJ. The CON-F in Newton per kilogram was calculated during the propulsive phase of the movement and normalized to body mass. V-peak is the peak velocity during the concentric phase. The eccentric phase (ECC) starts when the movement begins and ends when athlete is at the bottom of the loading phase of their jump.

### Sprint Tests

Sprinting performance was assessed using a sprint over a distance of 30 m and 60 m, adapting the protocol used by a previous study (Beckman and Tweedy, 2009). The sprint tests over 30 m and 60 m were performed by each subject starting with a free departure from a standing position. An electronic timing gates system (Globus^®^, Codogné, Italy) was used to record each performance time. Timing gates were set at 0.7 m above the ground and place 0.3 m back from the starting line. All participants performed three trials for each sprint test (s) and the best of those lowest were used for analysis.

### Sample Size Determination

The sample size was calculated based on power analysis [G^*^Power 3.1.9.2 (www.gpower.hlu.de/en.html)]. The analysis was based on desired $\eta_{\text{p}}^{2}$ of 0.14 (corresponding to a Cohen's *f* = 0.40, and to a Power (1–β err. Prob.) = 0.95) for the *F*-tests, and on desired ρ^2^ of 0.25 for the correlation analysis, which are interpreted as an index of large effect size (Cohen, 1988; Richardson, 2011). The analysis of the required sample size revealed that a number of 15 participants was satisfactory, as the effect size and observed power remained stable above this number. Likewise, this sample size was consistent with that obtained previously (Hatfield et al., 2006). These authors observed that a sample size ranging from 13 to 15 was adequate to highlight significant changes in MVC values after a strength training period.

### Statistical Analysis

The Shapiro-Wilk's test was conducted to verify if all data were normally distributed. An unpaired *t*-test was performed to assess potential differences at the baseline among variables. A two-way mixed analysis of variance (ANOVA) with a within-group (Time, pre, and post) and between-group (Group, SRT, and TRT) factors was performed to assess potential interactions (Time × Group) for each dependent variable. In case of significant differences in each of the variables at the baseline, an analysis of covariance (ANCOVA) was implemented to adjust for baseline conditions. As a measure of effect size for ANOVA, partial eta squared (pη^2^) was reported. The thresholds for a small, moderate and a large effect were defined as 0.01, 0.06, and 0.14, respectively (Cohen, 1988).

All the conventional statistical analyses were performed using the IBM SPSS^®^ Statistics software (v. 21, New York, U.S.A.). Data are shown as mean ± SD without covariate adjustments. An α value of 0.05 was set as criterion level of significance.

## Results

Tables 1, 2 show descriptive data and inferential statistics of pre- and post-training in the two groups. The participants did not present significant difference for several variables at the baseline (n.s.) except for isokinetic peak power of knee flexors at 60°/s, isokinetic peak power of knee flexors at 120°/s, isokinetic TW of knee flexors at 120°/s, and isokinetic peak power of knee extensors at 120°/s (*p* < 0.05).

**Table 1:** Results of the repeated measures ANOVAs (2×2) conducted on the countermovement jump (CMJ), total duration of the jump (Time), eccentric time (ECC-T), relative eccentric rate of force development (R-ECC-RFD), relative average vertical force during the concentric phase (CON-F), peak velocity in the concentric phase (V-peak), maximal isometric strength (MVC), sprint performance 30 m (30 m sprint) and 60 m (60 m sprint) using Time (Pre vs Post) and Group (Slow speed resistance training (SRT) vs Traditional resistance training (TRT) as factors.

	**Group**	**Pre**	**Post**	**Time × group**			**Time**			**Group**		
				***F*_**(1, 28)**_**	**$\eta_{\text{P}}^{2}$**	***p*-value**	***F*_**(1, 28)**_**	**$\eta_{\text{P}}^{2}$**	***p*-value**	***F*_**(1, 28)**_**	**$\eta_{\text{P}}^{2}$**	***p*-value**
CMJ (cm)	SRT	36.10 ± 2.47	38.70 ± 2.14	0.077	0.003	0.78	157.26	0.84	<0.0001	0.316	0.011	0.57
	TRT	35.67 ± 2.40	38.15 ± 2.66									
Time (ms)	SRT	435 ± 20	485 ± 21	2.466	0.11	0.127	39.46	0.72	<0.0001	0.394	0.015	0.53
	TRT	441 ± 33	471 ± 24									
ECC-T (ms)	SRT	243 ± 14	203 ± 16	0.143	0.08	0.60	63.22	0.89	<0.0001	9.17	0.62	<0.001
	TRT	261 ± 15	217 ± 12									
R-ECC-RFD (N.s-1.kg-1)	SRT	42 ± 7	50 ± 9	1.09	0.14	0.39	13.47	0.67	<0.01	0.27	0.08	0.67
	TRT	43 ± 9	49 ± 11									
CON-F (N.kg-1)	SRT	14.22 ± 1.6	19.46 ± 1.9	5.68	0.71	<0.01	51.64	0.84	<0.0001	2.16	0.41	0.09
	TRT	14.72 ± 1.8	17.35 ± 1.9									
V Peak	SRT	1.79 ± 0.09	1.91 ± 0.10	0.50	0.11	0.53	27.70	0.74	<0.0001	1.40	0.21	0.30
	TRT	1.78 ± 0.07	1.87 ± 0.11									
MVC (*N*)	SRT	41.71 ± 2.85	46.66 ± 2.59	9.485	0.25	<0.01	392.49	0.93	<0.0001	0.109	0.004	0.74
	TRT	40.49 ± 2.78	47.25 ± 2.61									
30 m sprint (s)	SRT	4.88 ± 0.35	4.59 ± 0.26	3.987	0.12	0.056	5.96	0.176	0.021	0.140	0.005	0.71
	TRT	4.71 ± 0.44	4.68 ± 0.25									
60 m sprint (s)	SRT	8.82 ± 0.64	8.48 ± 0.51	1.194	0.041	0.28	5.496	0.16	0.026	0.030	0.001	0.86
	TRT	8.68 ± 0.60	8.56 ± 0.47									

*Values are means ± SDs*.

**Table 2:** Results of the repeated measures ANOVAs (2×2) conducted on the maximal isometric strength (MVC), highest peak torque (Peak TQ) at 60°/s and 120°/s and the total work (TW) at 60°/s and 120°/s produced by flexors and extensors muscle on the dominant limb, using Time (Pre vs Post) and Group (Slow speed resistance training (SRT) vs Traditional resistance training (TRT) as factors.

		**Group**	**Pre**	**Post**	**Time × Group**			**Time**			**Group**		
					**F_**(1, 28)**_**	**$\eta_{\text{P}}^{2}$**	***p* value**	**F_**(1, 28)**_**	**$\eta_{\text{P}}^{2}$**	***p* value**	**F_**(1, 28)**_**	**$\eta_{\text{P}}^{2}$**	***p* value**
Kkee extensors	MVC (*N*)	SRT	41.71 ± 2.85	46.66 ± 2.59	9.485	0.25	0.005	392.49	0.93	<0.0001	0.109	0.004	0.74
		TRT	40.49 ± 2.78	47.25 ± 2.61									
	Peak TQ at 60 °/s (N·m)	SRT	139.01 ± 9.50	158.27 ± 8.76	0.714	0.025	0.40	358.15	0.92	<0.001	1.67	0.056	0.207
		TRT	144.03 ± 9.81	161.64 ± 8.96									
	Peak TQ at 120°/s (N·m)	SRT	83.03 ± 5.68	86.67 ± 4.79	11.955	0.30	0.002	13.63	0.33	0.001	2.248	0.077	0.14
		TRT	88.95 ± 6.05	89.05 ± 4.97									
	TW at 60 °/s (J)	SRT	366.26 ± 25.04	525.56 ± 29.10	143.60	0.83	<0.0001	3842.16	0.99	<0.0001	6.919	0.19	0.014
		TRT	353.54 ± 23.97	589.20 ± 32.63									
	TW at 120 °/s (J)	SRT	383.67 ± 26.23	444.59 ± 24.61	118.647	0.80	<0.0001	130.283	0.82	<0.0001	5.944	0.175	0.021
		TRT	392.24 ± 26.81	393.66 ± 31.80									
Knee flexors	Peak TQ at 60 °/s (N·m)	SRT	96.17 ± 6.57	98.01 ± 5.44	6.523	0.195	0.017	13.591	0.33	0.001	149.955	0.84	<0.0001
		TRT	91.09 ± 6.17	110.22 ± 6.10									
	Peak TQ at 120 °/s (N·m)	SRT	80.02 ± 5.47	86.06 ± 4.76	7.724	0.22	0.010	11.314	0.29	0.002	57.072	0.67	<0.0001
		TRT	73.97 ± 4.99	74.67 ± 4.48									
	TW at 60 °/s (J)	SRT	291.05 ± 19.90	315.64 ± 17.47	62.279	0.69	<0.0001	396.37	0.93	<0.0001	2.432	0.08	0.13
		TRT	285.21 ± 19.47	342.11 ± 19.92									
	TW at 120 °/s (J)	SRT	324.62 ± 22.19	372.19 ± 20.61	6.343	0.19	0.018	10.271	0.27	0.003	9.315	0.25	0.005
		TRT	342.87 ± 23.39	362.59 ± 24.21									

*Values are means ± SDs*.

### Training Protocol

TUT_50_ data recorded during training sessions shew, respectively, 268 ± 34 s for SRT and 247 ± 25 s TRT. No differences were found in TUT_50_ between training conditions [*t*(28) = 1.927, *p* = 0.064].

### Maximum Voluntary Contraction (MVC)

MVC: a significant interaction was observed [*F*_(1, 28)_ = 9.48, *p* = 0.005, pη^2^ = 0.25]. The main effect of the training condition was not significant [*F*_(1, 28)_ = 0.109, *p* = 0.74), whereas there was a significant main effect of time [*F*_(1, 28)_ = 392.49, *p* < 0.0001, pη^2^ = 0.93].

### Isokinetic Assessment

For knee extensors, no significant interaction was found in the Peak TQ at 60°/s (*p* = 0.405, pη^2^ = 0.025), whereas the effect of time was significant [*F*_(1, 28)_ = 358.15, *p* < 0.0001, pη^2^ = 0.92]. Furthermore, a significant interaction was found [*F*_(1, 28)_ = 11.95, *p* = 0.002, pη^2^ = 0.30] in the Peak TQ at 120°/s. A significant interaction was also found [*F*_(1, 28)_ = 143.6, *p* < 0.0001, pη^2^ = 0.83] in the TW at 60°/s, as well as in the TW at 120°/s [*F*_(1, 28)_ = 118.64, *p* < 0.0001, pη^2^ = 0.80]. For knee flexors, a significant interaction was found [*F*_(1, 28)_ = 6.52, *p* = 0.017, pη^2^ = 0.19] in the Peak TQ at 60°/s, as well as in the Peak TQ at 120°/s [*F*_(1, 28)_ = 7.722, *p* = 0.010, pη^2^ = 0.22]. Furthermore, significant interaction was found [*F*_(1, 28)_ = 62.27, *p* < 0.0001, pη^2^ = 0.69] in the TW at 60°/s, as well as in the TW at 120°/s [*F*_(1, 28)_ = 6.34, *p* = 0.018, pη^2^ = 0.19].

**Figure 2:**
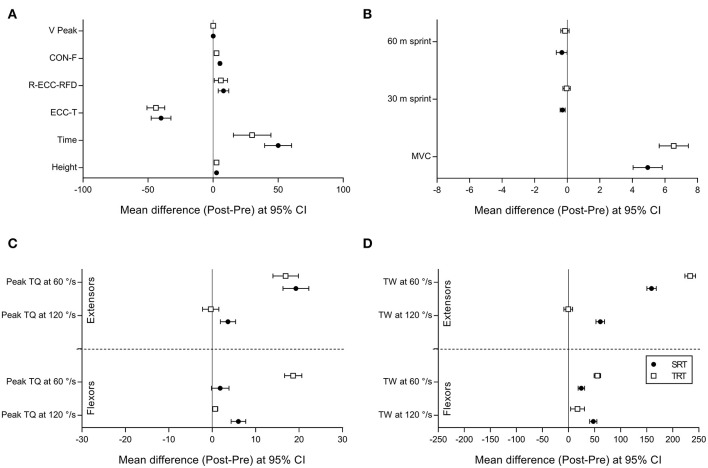
Graphical representation of the within-group changes (Post-pre difference at 95% CI) for SRT and TRT. **(A)** Jump height (Height) of countermovement jump (CMJ), peak velocity in the concentric phase (V-peak), relative average vertical force during the concentric phase (CON-F), relative eccentric rate of force development (R-ECC-RFD), eccentric time (ECC-T), total duration of the jump (Time). **(B)** Maximal isometric strength (MVC), sprint performance 30 m (30 m sprint) and 60 m (60 m sprint). **(C)** Isokinetic peak torque of knee extensors and flexors (Peak TQ) at 60 and 120°/s. **(D)** Isokinetic total work of knee extensors and flexors (TW) at 60 and 120°/s. SRT, Slow speed resistance training; TRT, Traditional resistance training.

A graphic representation of the within-group changes for physical performance and isokinetic parameters is provided in Figure 2.

### Jump Tests

The effect of the training condition for both groups on CMJ is shown in Table 1. For jump height (cm) a significant time effect was observed for time [*F*_(1, 28)_ = 157.26, *p* < 0.0001, pη^2^ = 0.84], whereas there was no significant main effect of the training condition [*F*_(1, 28)_ = 3.06, *p* = 0.09, pη^2^ = 0.099]. No significant interaction (Time^*^Training condition) was found (*p* = 0.784, pη^2^ = 0.03).

Similarly, for Time (ms) a significant time effect was observed [*F*_(1, 28)_ = 39.46, *p* < 0.0001, pη^2^ = 0.72]. There was no significant main effect for training [*F*_(1, 28)_ = 0.394, *p* = 0.53, pη^2^ = 0.01] and no significant interaction was found (*p* = 0.127, pη^2^ = 0.11).

No significant interaction was found in ECC-T (*p* = 0.143, pη^2^ = 0.08), a significant main effect for time [*F*_(1, 28)_ = 63.22, *p* < 0.0001, pη^2^ = 0.89] and training condition [*F*_(1, 28)_ = 9.17, *p* < 0.001, pη^2^ = 0.62] was observed. Tukey's *post hoc* analysis showed a significant difference between condition (groups) effects after training (T1) [*q*_(14)_ = 3.785, *p* < 0.05]. For R-ECC-RFD data no significant interaction was found (*p* = 0.39, pη^2^ = 0.14), a significant time effect was observed [*F*_(1, 28)_ = 13.47, *p* < 0.01, pη^2^ = 0.67], whereas there was no significant main effect of the training condition [*F*_(1, 28)_ = 0.27, *p* = 0.67, pη^2^ = 0.08]. A significant interaction was found for CON-F [*F*_(1, 28)_ = 5.68, *p* < 0.01, pη^2^ = 0.71], a significant main effect for time [*F*_(1, 28)_ = 51.64, *p* < 0.0001, pη^2^ = 0.84], whereas there was no significant main effect of the training condition [*F*_(1, 28)_ = 2.16, *p* = 0.09, pη^2^ = 0.41]. Tukey's *post hoc* analysis showed a significant difference between condition effects (groups) after training (T1) [*q*_(14)_ = 4.53, *p* < 0.05).

For V-peak, the main effect of the training condition was not significant [*F*_(1, 28)_ = 1.40, *p* = 0.30, pη^2^ = 0.21], whereas there was a significant main effect of time [*F*_(1, 28)_ = 27.70, *p* < 0.001, pη^2^ = 0.74]. No significant interaction (Time^*^Training condition) was found [*F*_(1, 28)_ = 0.50, *p* = 0.53, pη^2^ = 0.11].

### Sprint Tests

Regarding sprint performances, no significant interaction was found for 30 m (*p* = 0.056, pη^2^ = 0.12) and 60 m (*p* = 0.284, pη^2^ = 0.04), whereas the main effect of time was significant for 30 m [*F*_(1, 28)_ = 5.96, p = 0.021, pη^2^ = 0.17] and for 60 m [*F*_(1, 28)_ = 5.49, *p* = 0.026, pη^2^ = 0.16].

It should be noted that SRT, compared with TRT, exhibited a higher improvement (5.9 vs. 0.6%, respectively) in 30 m sprint, as also supported by the effect size ($\eta_{\text{P}}^{2}$ = 0.12; Figure 2).

## Discussion

The present study is the first time that long-term changes in contractile muscle force characteristics, in response to different types of speed-specific exercises training in elite futsal players, were examined. From a general perspective, both training modes were effective for improving the strength performance indices during the observed period. The two groups, however, presented remarkable differences in the within-group changes. SRT exhibited greater improvements in both Peak TQ and TW for knee extensors and flexors in isokinetic parameters at 120°/s (high velocity). A modification in counter movement jump strategies highlighted a decrease in ECC-T and an increase in CON-F compared to TRT group. Conversely, TRT showed greater improvements in MVC, and in both Peak TQ and TW for knee extensors and flexors at 60°/s (slow velocity), except for Peak TQ of the knee extensors, where no significant difference was found between TRT and SRT. All together these findings suggest that SRT induced a specific characterization of muscle rapid contraction capacity in high level athletes. We must point out that these adaptations have not led to significant greater improvements in the performance of functional tests such as jumping and sprinting compared to a traditional intervention. Nevertheless, a marked difference was found between the effects of the two interventions in the 30 m sprint (SRT + 5.9% vs. TRT + O.6%). Unfortunately, working with elite athletes makes it impossible to increase the sample size and ensure rigorous training procedures.

The present study supports the notion that ischemic condition, due to SRT, increases the fast-twitch fibers recruitment in contracting muscle (Takada et al., 2012a,b). According to Henneman's principle (Henneman et al., 1965), during low-intensity resistance training slow twitch fibers are prevalently recruited. However, several studies have shown that inadequate oxygen supply evokes early recruitment of fast-twitch fibers (Moritani et al., 1992; Takada et al., 2012b; Husmann et al., 2017).

Recently, Husmann et al. (2017) have clearly shown that limited blood flow condition accelerated the exercise-induced development of muscle fatigue (Husmann et al., 2017). Their finding described a strong decline in neuromuscular function due to peripheral (i.e., accelerated rate of phosphocreatine hydrolysis and inorganic phosphate accumulation) and neural (i.e., decreased motoneuron firing rates due to the inhibitory feedback of group III/IV muscle afferents) factors (Husmann et al., 2017). This higher muscular activation is communally interpreted as an augmented recruitment of fast twitch fibers (Pearson and Hussain, 2015).

For instance, a low-intensity (50% 1RM) resistance training session for knee extensor muscles, with sustained force generation, has been shown to suppress both blood inflow to, and outflow from, the muscle due to an increase in intramuscular pressure (Koba et al., 2004).

In support with this notion, we have found a specific increasing of rapid force contraction capacity (Peak TQ 120°/s and 30 m sprint) in SRT group. To the best of our knowledge, no data exist to sustain selective hypertrophy of fast twitch fibers in elite athletes in response to SRT in the literature. Moreover, the differences in Peak TQ generated at slow and high velocity in isokinetic test between SRT and TRT training show a specific characterization of muscle contractile capacity due to speed-specific exercise training. This is consistent with a recent study by Nielsen et al. (2017), who reported an increased rapid force capacity in subjects attending resistance training in blood flow-restricted condition (Nielsen et al., 2017). The authors, however, reported a delayed effect (12 days) in subjects gains in strength capacity due to slower recovery. Unfortunately, the assessment of delayed effects were beyond the aim of the present study. The faster adaptation and the consequent increased contractile performance found in the present study may be explained by the high fitness level of population recruited (Fernandez-Gonzalo et al., 2013).

The data from this study confirmed the suggestion made by Kim et al. (2011), who found a similar improvement in maximal muscle contraction in SRT and TRT groups (Kim et al., 2011). The significant main effect of time for MVC and Peak TQ reveal that both groups improved muscular strength over the training period (8 weeks). Conversely, MVC results are inconsistent with previous studies that reported greater increase for SRT group compared to TRT (Westcott et al., 2001; Schilling et al., 2008). Essentially, this contradictory finding is based on two underlying reasons: (i) training protocols in previous studies differed in duration, volume and the length of time that the muscle was under tension (Schilling et al., 2008); and (ii) observed population heterogeneity: the well-established effects on resistance training adaptations of gender (Westcott et al., 2001), age (Toji and Kaneko, 2007), experience with resistance training (Tanimoto and Ishii, 2006) and subjects fitness level (Fernandez-Gonzalo et al., 2013).

In this perspective, it is important to emphasize that in this study the training volumes of each session were calculated using the time under tension method with the aim of applying the same internal training load to both groups (Hatfield et al., 2006; Wilk et al., 2018, 2019). In contrast, in previous studies the traditional volumes training method was applied (i.e., load lifted multiplied number of repetition and sets; Kraemer and Ratamess, 2004). Therefore, our results support the notion that the length of time for which the muscle is under tension is a principal factor that affects improvement in muscle contractile force (Kim et al., 2011; Pearson and Hussain, 2015). In terms of limitations, we need to highlight that the present study has not accurately estimated the effective restricted blood flow inducing muscle oxygenation, and hormonal levels changes due to training. Further studies are therefore needed to corroborate our findings.

In accordance with the well-reported evidence of the literature, we assume that this increased contractile strength is likely because of the greater effect in muscle hypertrophy rather than improved neural activation (Tanimoto and Ishii, 2006). Moreover, systemic hormonal production (i.e., growth hormone) has been strongly associated with exercise under moderate hypoxic conditions (Takarada et al., 2000).

In summary, this study shows that a slow-speed training approach can be a very effective strategy in team-sports, especially in elite futsal players, where maximal acceleration and speed capacities play determinant roles in optimizing performance (Naser and Ali, 2016). The impact of our results on applied training science assume great importance in the light of the study by Chapman et al. (2011). They have reported reduced muscle damage during slow-speed exercise compared to fast-speed exercise (Chapman et al., 2011). Similar results have been observed recently, showing fast recovery without long-lasting impairments in motor performance after low-intensity blood flow restriction exercise (Husmann et al., 2017). It is interesting to note that both authors reported a decrease in post exercise muscular soreness perception (Chapman et al., 2011; Husmann et al., 2017; Iodice et al., 2019). The authors suggested that athletes would achieved greater compliance through slow-speed training (Chapman et al., 2011).

Moreover, the possibility of combining optimum training loads with reduced muscle damage emerges as a novel and suitable option for coaches and sport scientists, due to the applicability, efficiency and time-saving characteristics of SRT approach.

The outcomes of this research support the idea that slow-speed resistance training may allow a positive adaptation to futsal players' performance to be achieved. In team sports the training stimulus has been situated in a complex “load-balancing strategy” that frequently has to *sacrifice* the resistance training sessions to several factors, such as recovery time and DOMS. The reduced muscle damage could allow for an increase of SRT number of sessions to counteract the well-documented concurrent training effects between strength and endurance adaptations in team-sport training (Fyfe et al., 2014).

## Data Availability Statement

The datasets generated for this study are available on request to the corresponding author.

## Ethics Statement

The studies involving human participants were reviewed and approved by Ethics Committee of Institute of cognitive sciences and technologies, CNR, of Rome, Italy (N° 0003871). The patients/participants provided their written informed consent to participate in this study.

## Author Contributions

PI conceived and directed the study. DD directed all the training sessions and managed the workloads. PI, GP, and DD performed all functional measurement and tests. DF and AT performed data analysis. PI, DF, AT, and GA designed the training protocols. Finally, PI, DF, and AT wrote the manuscript.

### Conflict of Interest

The authors declare that the research was conducted in the absence of any commercial or financial relationships that could be construed as a potential conflict of interest.
